# Complement C5 inhibition with eculizumab or ravulizumab is associated with increased cardiovascular and thromboembolic risk after ST-elevation myocardial infarction: a propensity-matched global retrospective cohort study

**DOI:** 10.1186/s12872-026-05752-6

**Published:** 2026-03-23

**Authors:** Carl Vahldieck

**Affiliations:** 1 Department of Anesthesiology and Intensive Care Medicine, University Medical Centre Schleswig-Holstein Campus Luebeck, Luebeck, Germany; 2 Institute of Physiology, University of Luebeck, Luebeck, Germany; 3 DZHK (German Research Centre for Cardiovascular Research), Partner Site Hamburg/Luebeck/Kiel, Luebeck, Germany

**Keywords:** ST-Elevation-Myocardial-Infarction, Complement System, C5 Inhibition, Eculizumab, Ravulizumab, TriNetX Database, Propensity Score Matching

## Abstract

**Background:**

Complement-mediated myocardial and vascular injury is a hallmark of ST-elevation myocardial infarction (STEMI). Dysregulated activation of complement component C5 has been implicated in endothelial dysfunction, exacerbated inflammatory responses, and progressive vascular damage during acute ischemia. Experimental data suggest that antagonizing the C5a–C5aR1 axis may attenuate these effects, but clinical evidence regarding the impact of established C5 inhibitors during STEMI remains limited.

**Methods:**

To investigate real-world cardiovascular and thromboembolic outcomes associated with C5 inhibition during STEMI, a propensity score-matched retrospective cohort study using the TriNetX Global Collaborative Network was performed. 174 patients with STEMI who had received Eculizumab or Ravulizumab for pre-existing complement-mediated disorders (paroxysmal nocturnal hemoglobinuria, atypical hemolytic uremic syndrome, neuromyelitis optica, or myasthenia gravis) prior to the index event were compared with 174 matched patients without C5-inhibitor exposure. Outcomes were assessed at 30 days and 365 days after STEMI and included all-cause mortality, stroke or transient ischemic attack (TIA), arrhythmia, thrombotic disorders, major adverse circulatory events (MACE), acute kidney injury (AKI), and cardiomyopathy.

**Results:**

At 365 days, C5-inhibited patients demonstrated significantly higher risks of death (21.7% vs. 15.8; *p* < 0.05), stroke/TIA (12.9% vs. 6.8%; *p* < 0.05), thrombotic disorders (23.8% vs. 11.3%; *p* < 0.001), MACE (36.8% vs. 22.8%; *p* < 0.001), and AKI (61.1% vs. 22.5%; *p* < 0.001). Cardiomyopathy rates were comparable between groups. Arrhythmia occurred less frequently in the C5-inhibition cohort (30.3% vs. 37.4%; *p* < 0.05). Similar patterns were observed at 30 days, with significantly increased risks for thrombotic disorders, MACE, and AKI in the C5-inhibited group. Hazard ratios confirmed elevated risks for most cardiovascular and thromboembolic outcomes at both time points.

**Conclusion:**

In this global real-world cohort, prior treatment with Eculizumab or Ravulizumab was associated with increased mortality and heightened cardiovascular and thromboembolic risk following STEMI. These findings underscore the need for careful clinical consideration of ongoing C5-inhibitor therapy in the acute management of STEMI and highlight the importance of prospective studies to clarify the mechanistic and clinical implications of complement blockade in acute myocardial ischemia.

## Background

ST-Elevation-Myocardial-Infarction (STEMI) is an acute event of hypoxic myocardial necrosis leading to myocardial tissue damage accompanied by an excessive immune response activating several innate immune pathways including the complement system [1, 2]. Although complement activation is an important first-line mediator of inflammatory response, a dysregulated activation often enhances uncontrolled damage to the myocardium and vasculature and is associated with larger infarction size and poor clinical outcomes [3–5]. Particularly, the mechanisms underlying complement-mediated injury to the myocardium mediated by the late components of the complement system’s terminal pathway (C5 convertase and C5b-9) have previously been characterized [3, 4, 6]. The conversion of complement factor C5 into C5a and C5b is a crucial step in STEMI, which can lead to dysregulated complement activation [7]. C5a is considered the most potent pro-inflammatory anaphylatoxin and induces activation and polarization of lymphocytes and increased leukocyte adherence to endothelial cells [8–10]. During STEMI the C5a plasma concentrations can rise several fold causing local increases in blood flow, smooth muscle contraction, edema, cytokine storm, mast cell degranulation, and increased vascular permeability [2, 8, 10–12]. 

Several clinical trials have investigated complement inhibition in acute myocardial infarction. The CARDINAL (Complement And ReDuction of INfarct size after Angioplasty or Lytics) program included two phase II trials, COMMA (COMplement inhibition in Myocardial infarction treated with Angioplasty) and COMPLY (COMPlement inhibition in myocardial infarction treated with thromboLYtics), which evaluated the C5 inhibitor pexelizumab in patients receiving primary percutaneous coronary intervention or thrombolytic therapy [3, 4]. While these studies did not demonstrate a reduction in infarct size or consistent improvements in clinical outcomes, analyses from the CARDINAL program suggested a statistically significant reduction in mortality in certain patient subgroups. Similarly, the large APEX-AMI trial did not show a significant benefit of pexelizumab on major clinical outcomes in patients with STEMI undergoing primary PCI [13]. 

Unfortunately, no further clinical studies on C5 inhibition in the context of STEMI have been conducted in recent years, even though promising new drugs for complement inhibition are available. Eculizumab is a monoclonal antibody that inhibits complement protein C5, thereby preventing formation of the terminal complement complex (C5b-9). Over time it has received approvals for several rare, complement-mediated disorders such as Paroxysmal Nocturnal Hemoglobinuria (PNH) [14], Atypical Hemolytic Uremic Syndrome (aHUS) [15], Generalized Myasthenia Gravis (gMG) [16] and Neuromyelitis Optica Spectrum Disorder (NMOSD) [17]. Ravulizumab, also a C5 inhibitor, is a longer-acting molecule designed to reduce infusion frequency, among other benefits. It has many overlapping indications with Eculizumab, namely PNH, aHUS as well as gMG [18]. 

Both agents, Eculizumab and Ravulizumab, are approved therapies and are used in routine clinical practice for the treatment of rare complement-mediated disorders such as aHUS, gGM, NMOSD or PNH. Unfortunately, there are still no clinical studies that have tested whether Eculizumab/Ravulizumab therapy is beneficial in the context of STEMI.

These limitations underscore the need for real-world, longitudinal studies to better assess the long-term cardiovascular and thromboembolic risks associated with C5-inhibition via Eculizumab/Ravulizumab in patients with STEMI.

Despite extensive research on complement inhibition in acute myocardial infarction, clinicians lack evidence to guide the management of patients who experience STEMI while receiving established C5 inhibitor therapy. Previous STEMI trials focused on pexelizumab and showed no consistent benefit, and no prospective studies have evaluated eculizumab or ravulizumab during acute coronary events. Nevertheless, these agents are increasingly used for rare complement-mediated disorders, creating a clinically relevant population in whom myocardial infarction may occur under sustained C5 blockade. In the absence of feasible prospective trials, this real-world analysis addresses a critical knowledge gap by systematically evaluating cardiovascular and thromboembolic outcomes in this setting.

To explore whether a new double-blind clinical trial with approved C5 inhibitors, similar to the CARDINAL or APEX-AMI trials, would be informative, a large global longitudinal study using electronic health records (EHRs) from the TriNetX Global Collaborative Network was conducted. The database comprises EHRs from 157 Health Care Organization (HCOs) worldwide containing data on diagnoses in the form of ICD-10 codes, medications and medical procedures (see [19] for an extensive description of the data base). The Global Collaborative Network was chosen as it has large numbers of EHRs and a high degree of data coverage and completeness among the networks provided by TriNetX. It includes HCOs from 30 countries in North- and South-America, Europe, the Middle East, Africa, and the Asia–Pacific regions and uploads of EHRs are conducted daily by the individual HCOs. Several strategies for harmonization and integration of various data sources are implemented in the database.

The aim of the current study was to elucidate the risk of mortality as well as the risks of developing defined cardiac and vascular diseases among STEMI patients being treated with C5-inhibitors Eculizumab or Ravulizumab compared to a propensity-score matched control cohort without treatment.

## Methods

### Data source and methodological framework

This study was conducted as a retrospective, population-based cohort analysis on a global scale, incorporating propensity score matching (PSM) to address confounding. Electronic health records (EHR) were retrieved from the Global Collaborative Network of TriNetX to identify patients with a documented ST-elevation myocardial infarction (STEMI). Individuals were categorized according to prior exposure to the complement C5 inhibitors Eculizumab or Ravulizumab, prescribed for non-cardiac indications such as PNH, aHUS, gMG and NMOSD or to no such exposure. Using this design, retrospective cohort comparisons were performed to evaluate cardiovascular and thromboembolic outcomes as well as all-cause mortality. Outcome measures were defined a priori and operationalized using clinically relevant combinations of ICD-10 codes, selected in alignment with recent comparable investigations [20]. The full set of ICD-10 codes applied is summarized in Table 1.

**Table 1:** Definitions of clinical endpoints investigated in the study

Outcomes		ICD-10 codes
Mortality	All-cause death	R99
Stroke or TIA	- Transient cerebral ischemic attacks - Cerebral infarction	G45 I63
Arrhythmia	- Atrial fibrillation and flutter - Other cardiac arrhythmia - Paroxysmal tachycardia	I48 I49 I47
Thrombotic disorders	- Pulmonary embolism - Other venous embolism and thrombosis	I26 I82
Major Adverse Circulatory Events (MACE)	- Cerebral infarction - Subsequent ST elevation (STEMI) and non-ST elevation (NSTEMI) myocardial infarction - Cardiac arrest - Cardiogenic shock - Pulmonary embolism - Arterial embolism and thrombosis - Other venous embolism and thrombosis	I63 I22 I46 R57.0 I26 I74 I82
Acute Kidney Injury (AKI)	- Acute Kidney Failure	N17
Cardiomyopathy	- Ischemic cardiomyopathy - Unspecified diastolic (congestive) heart failure - Unspecified systolic (congestive) heart failure	I25.5 I50.30 I50.20

### Data acquisition and patient selection

Data extraction was performed between September 2025 and January 2026 from the TriNetX Global Collaborative Network, which was selected due to its extensive coverage and the high number of available EHRs. At the time of analysis, the network comprised data from approximately 188 million patients contributed by 157 HCOs worldwide. Eligible patients were adults aged 18 years or older with a recorded diagnosis of STEMI. Inclusion in the treatment cohort additionally required documented therapy with a C5 inhibitor (Eculizumab or Ravulizumab) for one of the predefined alternative indications. Patients whose STEMI index event occurred more than 20 years before data retrieval were excluded from the analysis.

The experimental cohorts were constructed as follows: The control cohort comprised patients over the age of 18 who had previously been diagnosed with one of the aforementioned diseases (PNH, aHUS, gMG or NMOSD) and who presented with acute STEMI. It is noteworthy that these patients had never received treatment with a C5 inhibitor of any kind. The treatment cohort also consisted of patients over the age of 18 with the aforementioned diseases and acute STEMI, but these patients had been treated with a C5 inhibitor (eculizumab or ravulizumab). Patients who had received any other form of drug-induced complement inhibition were excluded from the analysis.

For the control cohort, data were contributed by 113 healthcare providers, resulting in a final sample of 6379 patients meeting the STEMI diagnostic criteria (ICD-10: I21, I21.0, I21.1, I21.2, I22, I22.0, I22.1, I22.8, I22.9). After propensity score matching, the final analytical cohort comprised 174 patients in each group who fulfilled criteria for STEMI and prior administration of either Eculizumab (RXNORM: 591781; HCPCS: J1300; PCS: XW033C6, XW043C6) or Ravulizumab (RXNORM: 2107301; HCPCS: J1303, C9052), in conjunction with at least one qualifying comorbidity: PNH (D59.5), aHUS (D59.3), NMOSD (G36.0), or gMG (G70.0). Diagnostic categories (PNH, aHUS, NMOSD, gMG) are not mutually exclusive within the database and therefore do not necessarily sum to the total cohort size. Assignment to the treatment cohort was restricted to patients in whom the initial C5 inhibitor administration occurred within a defined temporal window ranging from three months before to one month prior to the STEMI index event. Given the rarity of indications for C5 inhibitor therapy, consideration of pharmacokinetic properties is warranted. Complete and sustained C5 blockade is not achieved immediately after treatment initiation and differs between agents. For eculizumab, full inhibition of free C5 is typically reached after completion of the induction phase, approximately four weeks after therapy initiation, with relevant interindividual variability [21]. In contrast, ravulizumab achieves immediate and sustained terminal complement inhibition after the first infusion due to its extended half-life [22]. Accordingly, an exposure window of one to three months prior to the index event was selected to ensure effective and comparable pharmacodynamic C5 inhibition and to minimize bias related to incomplete complement blockade [21, 22]. Pharmacodynamic studies demonstrate that terminal complement inhibition with C5 inhibitors is typically achieved rapidly after treatment initiation, supporting the assumption that patients treated within this exposure window were under sustained complement blockade at the time of the index event. The TriNetX platform records medication exposure but does not provide detailed dosing schedules or longitudinal laboratory parameters such as complement activity measurements (e.g., CH50), precluding direct assessment of treatment intervals or functional complement blockade.

### Confounder adjustment and propensity scoring

To mitigate confounding effects inherent to observational datasets, PSM was applied to align baseline characteristics between cohorts. This approach has been widely employed in observational cardiovascular research and may offer advantages compared with traditional multivariable regression techniques [23]. PSM was conducted separately for each sub-analysis. The covariate matrix incorporated demographic variables, including age at the index event, sex, and race. Race information in the TriNetX database is available only in aggregated categories (e.g., White, Black, Asian), and further subdivision into specific East Asian populations such as Japanese or Chinese patients is not provided within the dataset. Given their potential independent impact on cardiovascular outcomes, rare complement-mediated disorders (specifically PNH, aHUS, gMG and NMOSD) were explicitly integrated into the matching process. These diseases represent the primary clinical indications for complement C5 inhibitor therapy, and their inclusion in the propensity score model was intended to reduce confounding related to indication for treatment. The complete set of covariates comprised: age at index event, female sex, ethnicity, overweight and obesity (E66), diabetes mellitus (E08–E13), disorders of lipoprotein metabolism and other lipidemias (E78), essential (primary) hypertension (I10), chronic lower respiratory diseases (J40–J4A), hypothyroidism (E03.9), neoplasms (C00–D49), nicotine dependence (F17) or personal history of nicotine dependence (Z87.891), PNH (D59.5), aHUS (D59.3), NMOSD (G36.0), or gMG (G70.0). Following data retrieval, the ordering of rows within the covariate matrix was randomized. Propensity scores were estimated using logistic regression, and 1:1 matching was implemented via a greedy nearest-neighbor algorithm with a caliper of 0.1, using the scikit-learn library in Python [24]. Post-matching baseline characteristics were reassessed and are presented in Table 2. Continuous variables were assumed to be approximately normally distributed. Age was therefore reported as mean ± standard deviation, consistent with the statistical framework used for propensity score matching. Given the large sample size and the robustness of t-tests to moderate deviations from normality, parametric t-tests were used for group comparisons, and non-parametric tests were not required.

**Table 2:** Baseline characteristics before and after propensity-score matching

Characteristic	Before PSM C5 inhibition (*n* = 178)	Before PSM Control (*n* = 6379)	*p*-value	SD	After PSM C5 inhibition (*n* = 174)	After PSM Control (*n* = 174)	*p*-value	SD
Demographics								
Age at index, years	55.8 ± 17.5	68.6 ± 14.3	< 0.001	0.798	56.1 ± 17.6	56.6 ± 17.3	0.775	0.031
Female sex	109 (61.2%)	2869 (45.0%)	< 0.001	0.330	108 (62.1%)	108 (62.1%)	1.000	< 0.001
Underlying diagnoses								
Paroxysmal nocturnal hemoglobinuria (PNH)	11 (6.2%)	200 (3.1%)	0.023	0.145	11 (6.3%)	12 (6.9%)	0.829	0.023
Hemolytic uremic syndrome (aHUS)	117 (65.7%)	251 (3.9%)	< 0.001	1.704	113 (64.9%)	113 (64.9%)	1.000	< 0.001
Neuromyelitis optica spectrum disorder (NMOSD)	10 (5.6%)	200 (3.1%)	< 0.001	0.122	10 (5.7%)	10 (5.7%)	1.000	< 0.001
Generalized myasthenia gravis (gMG)	33 (18.5%)	2992 (46.9%)	< 0.001	0.634	32 (18.4%)	30 (17.2%)	0.779	0.030
Cardiovascular risk factors and comorbidities								
Overweight or obesity	63 (35.4%)	1617 (25.3%)	0.002	0.220	63 (36.2%)	58 (33.3%)	0.574	0.060
Chronic kidney disease (CKD)	48 (27.2%)	1218 (19.1%)	< 0.001	0.985	47 (27.1%)	46 (26.2%)	0.031	0.197
Diabetes mellitus	66 (37.1%)	2223 (34.8%)	0.538	0.046	66 (37.9%)	71 (40.8%)	0.583	0.059
Disorders of lipoprotein metabolism	90 (50.6%)	3387 (53.1%)	0.504	0.051	89 (51.1%)	90 (51.7%)	0.915	0.011
Essential hypertension	141 (79.2%)	4087 (64.1%)	< 0.001	0.341	137 (78.7%)	140 (80.5%)	0.690	0.043
Chronic lower respiratory diseases	58 (32.6%)	1876 (29.4%)	0.360	0.069	57 (32.8%)	60 (34.5%)	0.734	0.037
Hypothyroidism	33 (18.5%)	1260 (19.8%)	0.688	0.031	33 (19.0%)	38 (21.8%)	0.506	0.071
Neoplasms	82 (46.1%)	2744 (43.0%)	0.417	0.061	81 (46.6%)	88 (50.6%)	0.453	0.081
Nicotine dependence	41 (23.0%)	779 (12.2%)	< 0.001	0.287	39 (22.4%)	37 (21.3%)	0.795	0.028
Personal history of nicotine dependence	54 (30.3%)	1287 (20.2%)	< 0.001	0.235	54 (31.0%)	55 (31.6%)	0.908	0.012

Data are presented as mean ± standard deviation for continuous variables and as number (percentage) for categorical variables

*PSM* propensity score matching, *SD* standardized difference

### Outcome assessment and analytical strategy

The STEMI diagnosis date was defined as the index event. Outcome analyses were restricted to events occurring within 30 days and 365 days following the index event, with all pre-index outcomes excluded. These two follow-up intervals were selected to capture both early post-infarction outcomes and potential longer-term effects associated with chronic complement inhibition. Composite cardiac and vascular endpoints, along with all-cause mortality, were examined in both cohorts. Relative risks and risk differences were calculated, and hazard ratios (HR) for incident cardiovascular outcomes were estimated. The proportional hazards assumption was evaluated using the generalized Schoenfeld method integrated within the TriNetX platform. Where violations of this assumption were identified, HR were calculated separately for distinct time intervals. Statistical inference was based on 95% confidence intervals (CI). Survival probabilities were estimated using Kaplan–Meier methodology. To quantify the magnitude of observed differences between groups, effect sizes were expressed as Cohen’s d, with values of ≥ 0.20 to < 0.50 indicating small effects, ≥ 0.50 to < 0.80 medium effects, and ≥ 0.80 large effects. A p-value below 0.05 was considered indicative of statistical significance. Visualizations were generated using GraphPad Prism (GraphPad Software Inc., Boston, MA).

### Ethical considerations and data governance

All analyses were performed within the federated TriNetX research environment, which provides access exclusively to aggregated and de-identified patient data in accordance with the de-identification standards outlined in § 164.514(a) of the HIPAA Privacy Rule [25]. The study represents a secondary analysis of existing data and involves no direct interaction or intervention involving human participants. The de-identification process has been formally certified by a qualified expert as specified in § 164.514(b)(1) of the HIPAA Privacy Rule, with the most recent certification renewed in December 2020 [25]. The Ethics Committee of the University of Lübeck confirmed that no additional ethical approval is required for studies relying solely on TriNetX data. As no individual-level identifiers are available and no patient contact occurs, the requirement for informed consent is waived. This classification is consistent with prior publications from the University of Lübeck and the University Hospital Schleswig-Holstein [26–28], which similarly report that analyses of de-identified secondary data do not constitute human subject research. The University Hospital Schleswig-Holstein, Lübeck, is registered as a participating healthcare organization within the TriNetX network, ensuring compliance with all institutional, legal, and regulatory requirements. Reporting of the study followed the STROBE guidelines.

## Results

### Cohort description and patient characteristics

Prior to propensity-matching, two cohorts of STEMI patients were identified: (i) 178 STEMI-patients from 38 HCOs with C5-inhibition treatment due to pre-existing conditions such as PNH, aHUS, gMG or NMOSD (hereinafter referred to as the C5-inhibition cohort) and (ii) 6379 STEMI-patients from 113 HCOs without C5-inhibition treatment (hereinafter referred to as the control cohort). Two separate datasets were conducted: The first analysis included outcomes that occurred in the time window that started 1 day after the first occurrence of the index event (STEMI) and ended 365 days after the first occurrence of the index event. The second ended 30 days after the first occurrence of the index event. Endpoints were defined by clinically relevant combinations of ICD-10 codes selected prior to data collection in accordance with recent similar studies [20]. The ICD-10 codes used are represented in Table 1. Outcome analysis was performed on the cohorts after PSM. Baseline characteristics before and after propensity-score matching are shown in Table 2.

Patients with C5-inhibition were slightly younger with a mean age at index at 55.8 years (SD 17.5) vs. 68.6 years (SD 14.3) for controls (*p* < 0.001). A slight female predominance was noted (61.2% for C5-inhibition vs. 45.0% for controls; *p* < 0.001). PNH (*p* = 0.023), aHUS (*p* < 0.001), NMOSD (*p* < 0.001) and gMG (*p* < 0.001) were more frequent in the C5-inhibition group. Chronic kidney disease (CKD) was present in 27.2% of patients in the C5-inhibition cohort and 19.1% in the control cohort before propensity score matching, and remained comparable after matching (27.1% vs. 26.2%). Overweight and obesity (*p* = 0.002), Essential (primary) hypertension (*p* < 0.001) as well as Nicotine dependence or Personal history of nicotine dependence (both: *p* < 0.001) showed increased frequency in the C5-inhibition cohort. Patients categorized as Asian represented a small proportion of the study population, accounting for 4.0% of the C5-inhibition cohort and 3.87% of the control cohort.

To allow for better comparability, a stringent matching model (PSM) was employed and cases and controls were matched 1:1 for demographics and major diagnoses. Matching turned out statistically effective for all selected diagnoses. (Table 2.)

### Cardiovascular and thromboembolic events

Several cardiovascular and thromboembolic outcomes were considered. A composite endpoint was constructed for ischemic cerebral events (Stroke or TIA (Transient cerebral ischemic attacks). The term Arrhythmia was used to summarize atrial fibrillation and flutter, other cardiac arrhythmias or paroxysmal tachycardia. Thrombotic disorders consist of pulmonary embolism and other venous embolism and thrombosis. In order to estimate mortality related to cardiovascular and thromboembolic diseases, a composite endpoint reflecting Major Adverse Circulatory Events (MACE), including cerebral infarction, subsequent ST elevation (STEMI) and non-ST elevation (NSTEMI) myocardial infarction, cardiac arrest, cardiogenic shock, pulmonary embolism, arterial embolism and thrombosis and other venous embolism and thrombosis, was constructed. Acute kidney injury (AKI) or acute kidney failure was compared as a singular outcome variable. Cardiomyopathy includes Ischemic cardiomyopathy as well as unspecified systolic and diastolic (congestive) heart failure.

### Outcomes after 365-days post index event (STEMI)

**Fig. 1:**
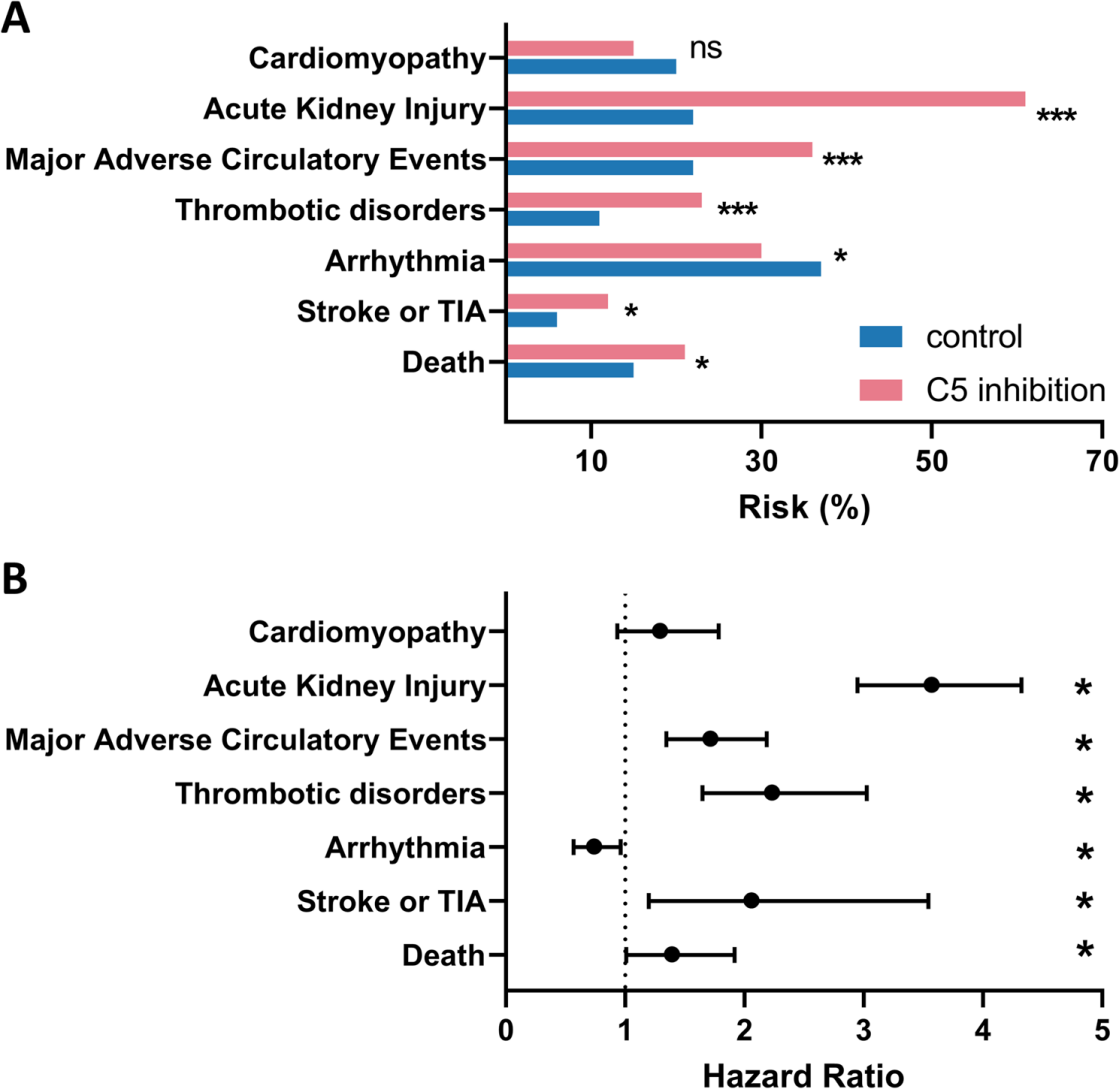
Cardiovascular and thromboembolic outcomes 365 days after STEMI in patients with and without C5 inhibition. **A** Bar graphs depict the 365-day prevalence of all-cause mortality, stroke or transient ischemic attack (TIA), arrhythmia, thrombotic disorders, major adverse circulatory events (MACE), acute kidney injury, and cardiomyopathy in patients treated with Eculizumab or Ravulizumab compared with propensity score–matched controls. **B** Corresponding hazard ratios with 95% confidence intervals derived from Cox proportional hazards models are shown for each endpoint. Asterisks indicate statistical significance levels (*p* < 0.05, *p* < 0.01, *p* < 0.001)

After 365-days (see Fig. 1A) patients in the C5-inhibition group exhibited a substantially higher prevalence of death relative to patients in the control group (21.7% vs. 15.8%; *p* = 0.033). Also Stroke or TIA occurred with higher prevalence in the C5-inhibition group (12.9% vs. 6.8%; *p* = 0.013). The Thrombotic Disorders were significantly higher in the C5-inhibition group after 365-days (23.8% vs. 11.3% *p* < 0.001). 36.8% of the C5-inhibition patients had a higher prevalence of MACE relative to 22.8% in the control group (*p* < 0.001). AKI occurred more frequently in the C5-inhibition group compared to patients in the control group (61.1% vs. 22.5%; *p* < 0.001). Cardiomyopathy (15.9% vs. 20.5%; not significant) did not differ significantly between groups. However, there were fewer cases of Arrhythmia in the in the C5-inhibition group than in the control cohort (30.3% vs. 37.4%; *p* = 0.048).

**Fig. 2:**
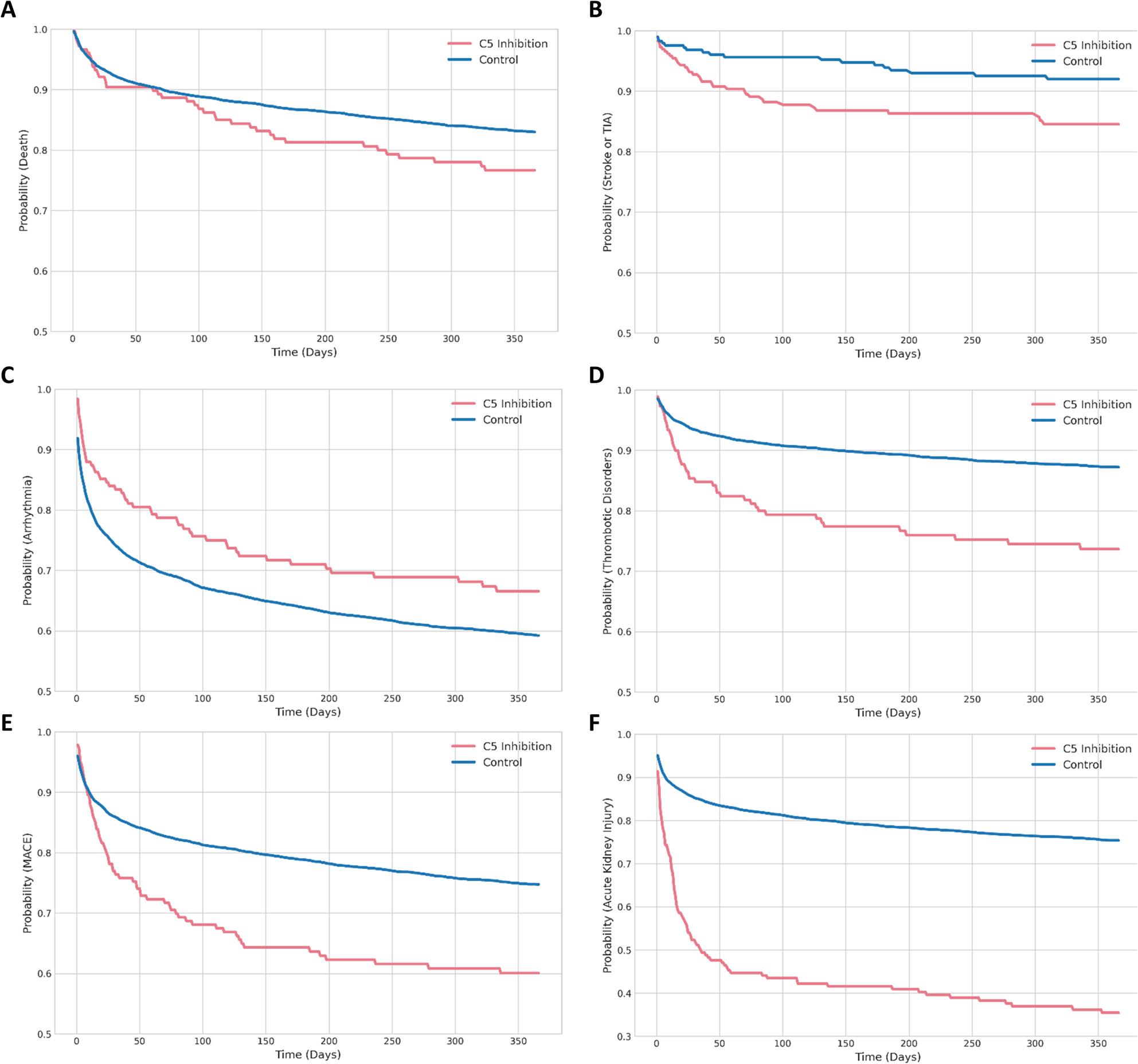
Kaplan–Meier estimates of 365-day cardiovascular and thromboembolic outcomes in STEMI patients with and without C5 inhibition. Kaplan–Meier curves display cumulative incidence probabilities over 365 days for **A** all-cause mortality, **B** stroke or transient ischemic attack (TIA), **C** arrhythmia, **D** thrombotic disorders, **E** major adverse circulatory events (MACE), and **F** acute kidney injury in patients treated with Eculizumab or Ravulizumab compared with propensity score–matched controls. Shaded areas denote 95% confidence intervals. Statistical comparisons are based on hazard ratios derived from Cox proportional hazards models

Overall, the HR for Death (*p* = 0.042), Stroke or TIA (*p* = 0.032), Thrombotic Disorders (*p* < 0.001), MACE (*p* < 0.001) and AKI (*p* < 0.001) were significantly higher in the C5-inhibition group compared to the control cohort (Fig. 1B; Table 3). Cardiomyopathy showed no significant value. The control group was associated with a significantly lower hazard for Arrhythmia (HR = 0.74; *p* = 0.041). To calculate probabilities regarding Death, Stroke or TIA, Arrhythmia, Thrombotic Disorders, MACE and AKI over time Kaplan-Meyer analysis were implemented comparing STEMI patients with C5-inhibition with matched controls (Fig. 2A-F).

**Table 3:** 365-days cardiovascular outcomes after STEMI with and without C5-inhibition showing risk ratio, hazard ratio and risk difference with 95%-confidence intervals (CI) and effect size (Cohen’s D)

**Outcome**	**Control (%)**	**C5-Inhibition (%)**	**Risk Ratio (95% CI)**	**Hazard Ratio (95% CI)**	**Risk Difference (95% CI)**	**z**	***p*** **-value**	**Cohen’s D**
Death	15.8%	21.7%	1.373 (1.034, 1.823)	1.392 (1.011, 1.917)	0.059 (-0.002, 0.120)	2.129	**0.033**	0.17
Stroke or TIA	6.8%	12.9%	1.900 (1.133. 3.186)	2.061 (1.199. 3.544)	6.1% (1.3%. 11.0%)	2.5	**0.013**	0.35
Arrhythmia	30.3%	37.4%	0.810 (0.649, 1.010)	0.739 (0.567, 0.963)	-0.071 (-0.138, -0.004)	-1.978	**0.048**	-0.12
Thrombotic disorders	11.3%	23.8%	2.104 (1.612, 2.745)	2.232 (1.648, 3.025)	0.125 (0.063, 0.187)	5.223	**<0.001**	0.41
MACE	22.8%	36.8%	1.613 (1.329, 1.958)	1.716 (1.346, 2.187)	0.140 (0.070, 0.210)	4.445	**<0.001**	0.26
Acute kidney	22.5%	61.1%	2.715 (2.401, 3.071)	3.571 (2.949, 4.324)	0.386 (0.315, 0.457)	12.213	**<0.001**	0.55
Cardiomyopathy	20.5%	15.9%	1.290 (0.966, 1.721)	1.292 (0.935, 1.785)	0.046 (-0.013, 0.105)	1.687	0.092	0.14

*MACE* Major Adverse Circulatory Events, *CI* Confidence interval, *TIA* Transient ischemic attacks, Boldface *p*-values indicate statistical significance

### Outcomes after 30-days post index event (STEMI)

**Fig. 3:**
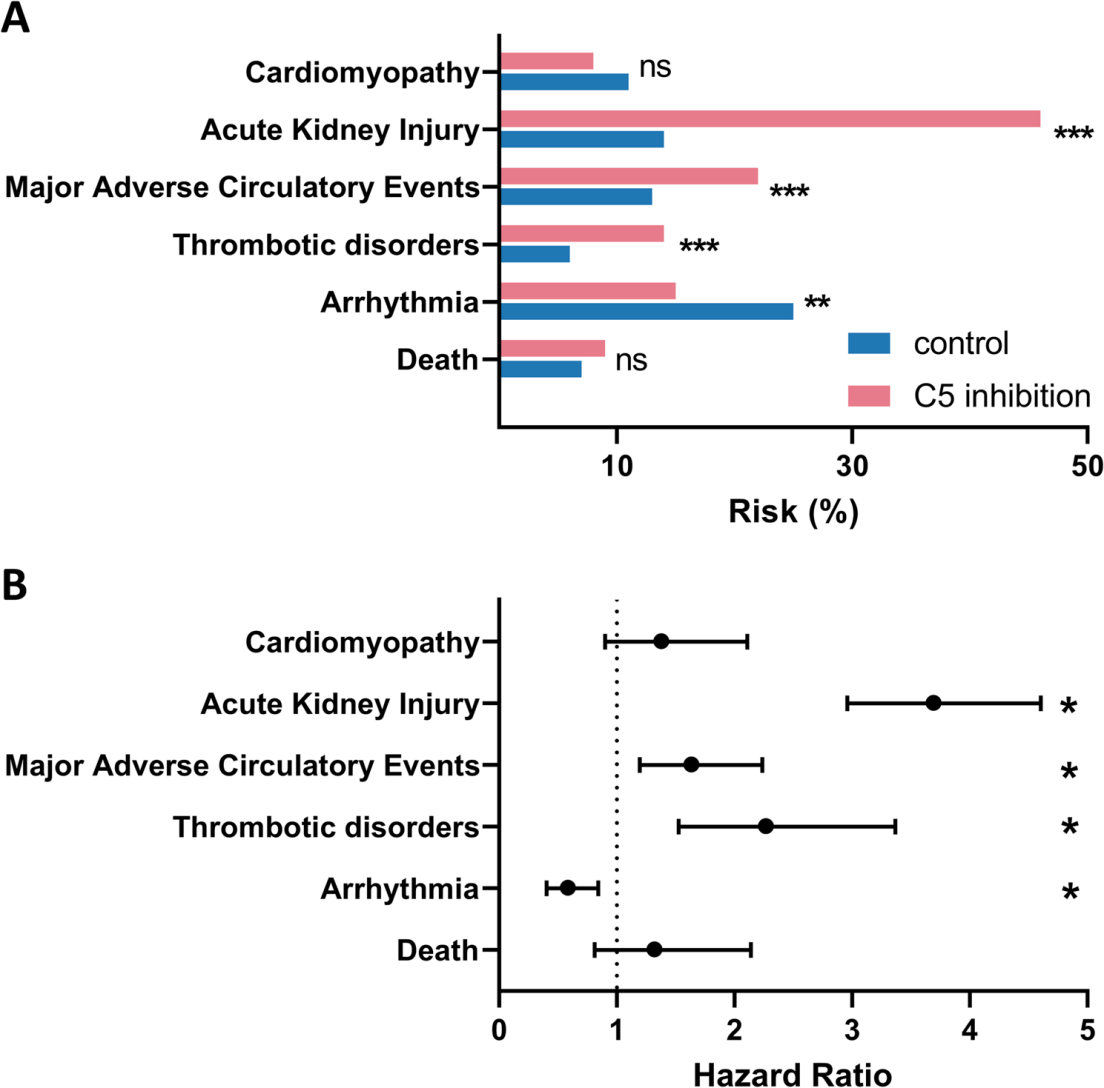
Cardiovascular and thromboembolic outcomes 30 days after STEMI in patients with and without C5 inhibition. **A** Bar graphs present the 30-day prevalence of all-cause mortality, arrhythmia, thrombotic disorders, major adverse circulatory events (MACE), acute kidney injury, and cardiomyopathy in STEMI patients treated with Eculizumab or Ravulizumab compared with matched controls. **B** Corresponding hazard ratios with 95% confidence intervals derived from Cox proportional hazards models are provided for each outcome. Asterisks denote statistical significance (*p*<0.05, *p*<0.01, *p*<0.001)

Compared with matched controls, patients treated with C5-inhibition exhibited a substantially higher prevalence of Thrombotic Disorders (15.7% vs.24.9%; *p* < 0.01), MACE (22.2% vs. 13.6%; *p* < 0.001), and AKI (40.3% vs. 17.3%; *p* < 0.001) (Fig. 3). By contrast, the prevalence Cardiomyopathy (9.2% vs. 7.1%) did not differ significantly between groups (Fig. 2A). There were fewer cases of Arrhythmia in the C5-inhibition cohort compared to untreated controls (15.7% vs. 24.9%; *p* = 0.004).

**Fig. 4:**
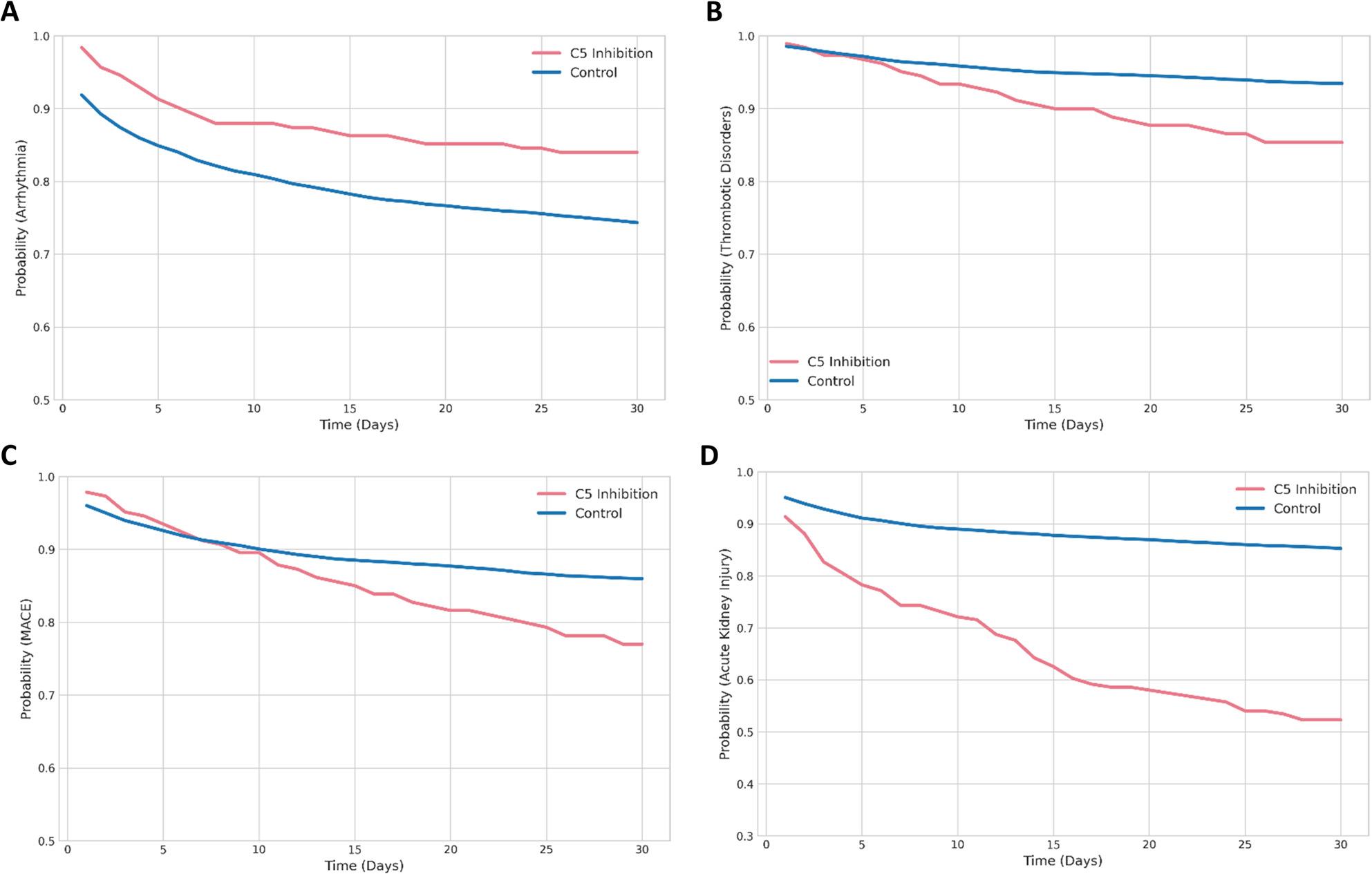
Kaplan–Meier estimates of 30-day cardiovascular and thromboembolic outcomes in STEMI patients with and without C5 inhibition. Kaplan–Meier curves illustrate cumulative incidence probabilities over 30 days for **A** all-cause mortality, **B** thrombotic disorders, **C** major adverse circulatory events (MACE), and **D** acute kidney injury in patients treated with Eculizumab or Ravulizumab compared with propensity score–matched controls. Shaded areas indicate 95% confidence intervals. Hazard ratios were computed using Cox proportional hazards modelling

Overall, the HR for Death (*p* < 0.01), Thrombotic Disorders (*p* < 0.01), MACE (*p* < 0.001) and AKI (*p* < 0.001) were significantly higher in the C5-inhibition group compared to the control cohort (Fig. 3; Table 4). Death and Cardiomyopathy showed no significant values 30 days after the index event. Probabilities regarding Arrhythmia, Thrombotic Disorders, MACE and AKI over time were conducted by Kaplan-Meyer analysis comparing STEMI patients with C5-inhibition with matched controls (Fig. 4A-D).

**Table 4:** 30-days cardiovascular outcomes after STEMI with and without C5-inhibition showing risk ratio, hazard ratio and risk difference with 95%-confidence intervals (CI) and effect size (Cohen’s D)

**Outcome**	**Control (%)**	**C5-Inhibition (%)**	**Risk Ratio (95% CI)**	**Hazard Ratio (95% CI)**	**Risk Difference (95% CI)**	**z**	***p*** **-value**	**Cohen’s D**
Death	7.1%	9.4%	1.329 (0.838, 2.107)	1.320 (0.813, 2.141)	0.023 (-0.020, 0.067)	1.200	0.230	0.16
Arrhythmia	24.9%	15.7%	0.630 (0.450, 0.882)	0.585 (0.405, 0.844)	-0.092 (-0.145, -0.039)	-2.867	**0.004**	-0,26
Thrombotic disorders	6.3%	14.1%	2.239 (1.550, 3.234)	2.268 (1.527, 3.369)	0.078 (0.027, 0.128)	4.239	**<0.001**	0.44
MACE	13.6%	22.2%	1.634 (1.240, 2.155)	1.636 (1.197, 2.237)	0.086 (0.026, 0.146)	3.350	**<0.001**	0.27
Acute kidney	14.3%	46.5%	3.247 (2.753, 3.830)	3.692 (2.961, 4.603)	0.322 (0.249, 0.394)	12.042	**<0.001**	0.64
Cardiomyopathy	11.9%	8.5%	1.396 (0.936, 2.083)	1.379 (0.901, 2.110)	0.034 (-0.013, 0.081)	1.616	0.106	0.18

*MACE* Major Adverse Circulatory Events, *CI* Confidence interval, Boldface *p*-values indicate statistical significance

## Discussion

The aim of the current study was to evaluate the potential benefits of a C5-inhibition in STEMI. Although a number of approaches have been taken in this direction, including the APEX-AMI, COMMA and COMPLY trials [3, 4, 13], further research is required in this area. Despite the fact that certain endeavors have been undertaken in this particular direction, there are currently no major clinical studies investigating the effects of Eculizumab or Ravulizumab on cardiovascular outcomes in the context of STEMI. The present study has achieved this objective through the implementation of a propensity score-matched cohort study.

The data presented here clearly indicate a significantly increased risk profile for cardiovascular and thrombogenic events in patients taking either Eculizumab or Ravulizumab and experience acute myocardial infarction. Although the study population was younger than typical cohorts reported in large STEMI trials, the included patients were affected by severe complement-mediated systemic diseases such as aHUS and PNH. These conditions are associated with endothelial dysfunction, thrombotic complications, and multi-organ involvement, which may substantially increase baseline cardiovascular risk and contribute to the relatively high mortality observed in this real-world cohort [5, 29, 30]. In addition, patients with complement-mediated disorders frequently present with significant systemic comorbidities, including renal dysfunction, thrombotic microangiopathy, and chronic inflammatory vascular injury, which may further increase mortality risk beyond that observed in conventional STEMI populations. The risk of mortality and the risk of developing certain cardiovascular diseases were significantly increased in STEMI patients treated with the C5 inhibitors Eculizumab or Ravulizumab compared to a propensity score-matched control cohort without treatment.

After both study periods (30 days and 365 days), the overall risk of mortality, ischemic cerebral events, thrombotic events, Major Adverse Cardiovascular Events, and acute renal failure was significantly increased in the treatment cohort.

However, there are some limitations to this study. First, this analysis is a retrospective evaluation of patient data from various HCOs. Accordingly, the data sets were not fully available, which limited the possibilities for deeper analysis. Thus, unfortunately it was not possible to perform subgroup analyses for each individual pre-existing disease (PNH, aHUS, NMOSD, gMG) in order to investigate the effects of the underlying disease on the observed negative outcomes. Second, due to the narrow therapeutic window for C5 inhibitors, only a select group of patients could be studied. Furthermore, detailed treatment schedules and laboratory markers of complement inhibition, such as CH50 levels, were not available within the TriNetX database, preventing direct confirmation of dosing regularity and sustained functional complement blockade. Detailed clinical indicators of disease severity, such as laboratory markers of hemolysis (e.g., LDH), transfusion requirements, or hospitalization frequency, were not consistently available within the TriNetX dataset, limiting the possibility of constructing a composite disease severity score.

Patients with aHUS constitute a particularly vulnerable subgroup, as active thrombotic microangiopathy is frequently associated with AKI, systemic endothelial dysfunction, and occasional cardiac involvement, all of which may increase short-term cardiovascular risk. The relatively high proportion of aHUS diagnoses within the treatment cohort likely reflects the fact that aHUS represents one of the most frequent clinical indications for long-term complement C5 inhibition in routine practice. While the exposure window was chosen to ensure complete C5 blockade, the retrospective EHR structure did not allow reliable distinction between acute TMA-related organ injury and chronic disease, nor precise temporal attribution of prior cardiac events. Although renal and cardiovascular comorbidities prior to the index event were balanced by PSM, residual confounding related to disease activity cannot be fully excluded [31–33]. Genetic information regarding complement pathway mutations in patients with aHUS was not available in the TriNetX database. Consequently, stratification according to the underlying genetic etiology of aHUS, which may influence thrombotic and cardiovascular risk, was not possible in the present analysis.

These were specifically patients who received C5 inhibitor therapy due to pre-existing diseases like PNH, aHUS, NMOSD or gMG. Furthermore, only patients who had received a full course of therapy with one of the C5 inhibitors were included in the study. Since anti-C5 treatment does not immediately lead to complete complement blockade [21], and in order to avoid systematic temporal bias, the treatment window was defined in such a way that complete C5 inhibition was ensured in all patients. Recent pharmacodynamic analyses highlight substantial differences between eculizumab and ravulizumab with respect to onset, depth, and stability of terminal complement inhibition, emphasizing that incomplete or fluctuating C5 blockade may occur during early treatment phases, particularly with eculizumab [34]. Phase 3 data show that Ravulizumab achieves complete and sustained C5 inhibition at the end of the first infusion [22], with steady-state concentrations reached after approximately two weeks [22]. In contrast, complete blockade with Eculizumab usually only occurs during the induction phase after approximately one month [21]. To ensure complete therapy with both medications, the therapeutic window was set at 3 to 1 month before the index event.

Although the TriNetX database contains several million health-records, this analysis included only a relatively small number of patients due to strict inclusion and exclusion criteria. In addition, ethnicity data within the TriNetX platform are limited to broad categories, precluding a more detailed analysis of specific East Asian populations such as Japanese or Chinese patients.

Nevertheless, analysis of real-world data is currently the best way to investigate the potential benefits of C5 inhibition in the context of STEMI, as there are no convincing clinical trials that have investigated clinical outcomes in the context of acute STEMI under Eculizumab or Ravulizumab therapy.

Most clinical studies on complement C5 inhibition in acute myocardial infarction have been conducted with Pexelizumab (another C5-targeted antibody fragment) and large clinical trials (e.g., APEX-AMI/COMPLY) [3, 4, 13] showed no clinical benefit. Complement inhibition has also been evaluated in cardiac surgical settings, most notably with pexelizumab during cardiopulmonary bypass, where combined analyses from the PRIMO-CABG I and II trials suggested a potential reduction in short-term mortality in high-risk patients, although the primary composite endpoint was not significantly improved, highlighting differences in pathophysiological drivers of complement activation between surgery and acute myocardial infarction [35, 36]. For Eculizumab and Ravulizumab, only preclinical data, rational considerations, and case reports are available, but no large-scale randomized studies investigating routine use in STEMI.

The present study showed that the risk of mortality and the risk of certain cardiovascular diseases were significantly increased in STEMI patients treated with C5 inhibitors. However, as this is a retrospective cohort analysis, the mechanistic background can hardly be satisfactorily explained.

In a mouse model of myocardial ischemia and reperfusion injury Busche et al. showed, that systemic inhibition of C5 (with antibody BB5.1) 30 min prior to reperfusion ensured better heart function, less troponin I (less cell damage), and less neutrophil infiltration [12]. Recent experimental data further demonstrate that myocardial infarction is associated with dynamic regulation of complement components C3 and C5 as well as sustained upregulation of C5aR1 signaling in infarcted tissue, underscoring the persistent involvement of terminal complement pathways beyond the acute ischemic phase [37]. This shows that without C5 activation, there is less acute damage in I/R. This fits with the idea that complement could be harmful during STEMI and lead to greater ischemia and reperfusion injury [5]. In human heart tissue from fresh and old infarcts, upregulation of mRNA and proteins of complement factors (C1q, C3, C5, etc.) as well as deposition of C4d, C3d, MAC (C5b-9) on damaged cardiomyocytes was demonstrated. This proves that complement activation occurs after myocardial infarction in humans, supporting mechanisms involving complement-mediated cell damage or inflammation [38]. Furthermore, patients with acute myocardial infarction had elevated plasma levels of C3d, C4d, Bb, and SC5b-9 compared to angina pectoris/controls. This indicates systemic complement activation in acute infarctions as these markers correlate with the extent of damage and poorer prognosis [39]. Complement component 3 has been shown to play an important role in preserving myocardial structure and function during the chronic remodeling phase after myocardial infarction [40]. In a mouse model, permanent loss of C3 had no effect on acute cell death after LAD ligation. However, it led to poorer heart function and more structural remodeling in the chronic phase. In addition, there was also less activation of cardiac progenitor/stem cells. Complement appears to be important for the long-term healing and remodeling phase. The study by Wysoczynski et al. shows that complement can also have positive effects on tissue repair and maintenance [40]. During reperfusion, complement contributes to both acute tissue damage (inflammation, cell lysis) and subsequent cell clearance. This means that C5 blockade can reduce acute I/R damage (positive effects in animal models), but at the same time disrupt the balance of healing processes [41]. Complement products (especially C3/C3a signaling) are important for the recruitment/activation of repair cells and for physiological scar remodeling. Some animal studies (C3 knockout) have shown poorer long-term function/impaired repair, i.e., comprehensive blockade of the terminal complement cascade may be detrimental in the long term [40, 42]. Complement products interact with endothelium and platelets, and modulation can influence platelet function, fibrinolysis, and plaque inflammation [5, 30, 43]. 

There are no large, direct studies investigating Ravulizumab in acute myocardial infarction that clearly show an increased risk of mortality. Statements regarding risk are therefore based on biological plausibility, animal data, observations on C5 inhibitors in general (e.g., Pexelizumab trials), and safety profiles from other indications (e.g., PNH or gMG) [44]. 

The negative outcomes presented in this study appear to be more related to vascular involvement (acute renal failure, severe circulatory disorders, thrombotic diseases) and seem to have little influence on the repair mechanisms of the myocardium (no increased risk of heart failure and even a reduction in the risk of cardiac arrhythmias). In line with this observation, recent human data indicate that increased expression of C5aR1 on circulating platelets correlates with coronary artery disease severity and prothrombotic platelet phenotypes, linking complement activation directly to thrombotic risk [45]. Further, these complications could be caused by a lack of C5a-mediated macrophage activation. After acute myocardial infarction, activation of the complement system, particularly via the C5a-C5aR1 axis, leads to pronounced activation of macrophages and endothelial cells, which exacerbates local inflammation and tissue damage [7, 46]. Animal studies show that C5aR1-deficient mice have smaller infarct sizes and improved cardiac function, confirming the pathogenic role of C5a signaling in cardiac repair [47]. While selective C5a receptor (C5aR1) blockade has shown cardioprotective effects in experimental models of myocardial ischemia–reperfusion injury, including reduced infarct size and inflammation, no prospective or real-world clinical data exist evaluating approved C5aR1 inhibitors in patients with STEMI [47]. 

In addition, C5a contributes to damage to the endothelial glycocalyx and impairment of vascular function, indicating a significant vascular component of the C5a-mediated immune response [7, 48]. 

Clinical trials with the C5 inhibitor pexelizumab showed a reduction in certain inflammatory markers, but no consistent benefit for mortality or cardiac events after acute infarction [13]. 

At the same time, there was no evidence that C5 blockade primarily exacerbates vascular complications, although thrombotic events in other clinical contexts are not completely prevented under C5 inhibition [29]. 

Overall, these findings suggest that C5a-mediated macrophage activation drives both cardiac and vascular damage after ischemic events, while the clinical translation of C5 blockade remains limited due to inconsistent results [7, 13, 29]. Contemporary reviews emphasize that C5a–C5aR1 signaling exerts context-dependent effects, acting as a driver of acute inflammatory and vascular injury while simultaneously contributing to immune regulation and tissue remodeling, thereby complicating the clinical translation of sustained C5 blockade [49]. Although proximal complement inhibition with the C3 inhibitor pegcetacoplan is approved and effective in PNH and shows clinical benefits in C3 glomerulopathy [50], no clinical or real-world data currently exist assessing C3 inhibition in STEMI patients, precluding conclusions about its cardiovascular impact.

Finally, all these studies show that targeted C5 inhibition does not simply have only positive effects in acute myocardial infarction. The lack of effect on infarct size shown in COMMA and COMPLY contradicts some theoretical advantages as fluctuating effects on mortality show that risks and side effects are also possible or that benefits are highly context-dependent.

Several important limitations inherent to the present study design should be acknowledged. First, the treatment cohort may initially appear highly artificial, as C5 inhibitors are prescribed almost exclusively for rare complement-mediated disorders such as PNH, aHUS, NMOSD, or gMG. However, this specificity represents a central strength of the study: the rarity of this patient population makes a prospective or randomized design virtually impossible, and real-world evidence therefore provides the only feasible approach to investigate cardiovascular risk in this setting. Although residual confounding related to the underlying diseases cannot be completely excluded, all major diagnoses were incorporated into the propensity score model to minimize imbalance between groups as rigorously as possible. In addition, treatment with complement C5 inhibitors is typically initiated based on disease activity and clinical severity in conditions such as PNH, aHUS, gMG, and NMOSD. Consequently, patients receiving C5 inhibition may represent a population with more advanced or clinically active underlying disease, which could influence baseline risk profiles independently of complement blockade.

Second, the relatively small sample size mirrors the current epidemiology of patients simultaneously affected by both C5-inhibitor therapy and STEMI. The number of individuals meeting these criteria worldwide remains extremely limited. Nevertheless, as the indications and clinical use of C5 inhibitors continue to expand, this population is expected to grow, increasing the clinical relevance of understanding their cardiovascular risk profile. Third, the depth of available clinical information was constrained by the structure of the TriNetX database, which does not provide complete datasets for all patients and therefore limits the feasibility of detailed subgroup analyses or adjustment for STEMI-specific parameters such as infarct size, revascularization strategy, or procedural timelines. In addition, detailed STEMI-related procedural variables, such as reperfusion strategy, door-to-balloon time, or thrombolytic therapy timing, were not consistently available within the TriNetX database. Given the observational design and the absence of detailed STEMI procedural variables within the database, the present findings should be interpreted as associations rather than evidence of a causal effect of C5 inhibition on post-infarction outcomes. These factors are known to substantially influence outcomes after myocardial infarction and therefore could not be accounted for in the present analysis.

The exposure definition in this study reflects established C5 inhibitor therapy prior to the STEMI index event rather than verified continuous treatment throughout the entire follow-up period. Because the TriNetX platform captures medication exposure but does not consistently provide longitudinal adherence or discontinuation data across HCOs, sustained complement blockade during follow-up cannot be directly confirmed at the individual patient level. The present analysis therefore evaluates cardiovascular outcomes in patients with documented pre-existing complement inhibition at the time of STEMI. Despite these limitations, the present study offers important and timely insights, particularly given the absence of prospective clinical data evaluating the impact of C5 inhibition in acute myocardial infarction. These findings highlight a critical knowledge gap and underscore the need for further mechanistic and clinical investigation as the use of complement-targeted therapies increases.

The data presented show that treatment with Eculizumab or Ravulizumab unfortunately has no positive effect on the cardiovascular outcome of STEMI patients. The risk profile presented here for both short-term (30 days) and long-term (1 year) outcomes should definitely be taken into account for future clinical studies.

## Conclusion

In this global propensity score–matched cohort study, prior treatment with the C5 inhibitors Eculizumab or Ravulizumab was associated with a substantially increased risk of mortality, thrombotic events, MACE, cerebrovascular complications, and AKI following STEMI. These adverse outcomes were consistent across both short-term and long-term follow-up periods, whereas cardiomyopathy rates remained comparable and arrhythmia risk was lower in the C5-inhibited cohort. Taken together, these findings indicate that systemic C5 blockade does not confer cardioprotective effects in the setting of acute myocardial infarction and may instead exacerbate vascular and thromboembolic vulnerability during the post-infarction period.

Importantly, the findings of the present study should not be interpreted as evidence to discontinue established C5 inhibitor therapy in patients with acute myocardial infarction. In individuals with complement-mediated disorders such as aHUS or PNH, interruption of C5 inhibition may precipitate disease exacerbation or relapse, particularly in the setting of acute inflammatory or ischemic stress. Given the absence of prospective clinical trial data and the observational nature of the present analysis, treatment decisions in this context should remain individualized and guided by multidisciplinary clinical judgment rather than by premature changes in established complement-targeted therapy.

Given the growing use of C5 inhibitors for rare complement-mediated disorders, awareness of these risks is essential for clinicians managing STEMI in this patient population. Future prospective studies are warranted to elucidate the mechanisms underlying these observations, to clarify the interaction between complement inhibition and ischemia–reperfusion biology, and to define evidence-based strategies for the acute and longitudinal care of STEMI patients receiving complement-targeted therapies.

## Data Availability

The dataset examined in this study is available upon reasonable request from the corresponding author.

## Abbreviations

aHUS: Atypical hemolytic uremic syndrome
AKI: Acute kidney injury
CARDINAL: Complement And ReDuction of INfarct size after Angioplasty or Lytics program
CI: Confidence interval
COMMA: Complement inhibition in myocardial infarction treated with angioplasty trial
COMPLY: Complement inhibition in myocardial infarction treated with thrombolytics trial
EHR: Electronic health record
gMG: Generalized myasthenia gravis
HCO: Health care organization
HR: Hazard ratio
ICD-10: International Classification of Diseases, 10th Revision
MACE: Major adverse circulatory events
NMOSD: Neuromyelitis optica spectrum disorder
PNH: Paroxysmal nocturnal hemoglobinuria
PSM: Propensity score matching
STEMI: ST-elevation myocardial infarction
STROBE: Strengthening the Reporting of Observational Studies in Epidemiology
TIA: Transient ischemic attack
